# A GWAS for grip strength in cohorts of children—Advantages of analysing young participants for this trait

**DOI:** 10.1111/gbb.70003

**Published:** 2024-10-08

**Authors:** Filippo Abbondanza, Carol A. Wang, Judith Schmitz, Krzysztof Marianski, Craig E. Pennell, Andrew J. O. Whitehouse, Silvia Paracchini

**Affiliations:** ^1^ School of Medicine University of St Andrews St Andrews Scotland; ^2^ School of Medicine and Public Health University of Newcastle Callaghan New South Wales Australia; ^3^ Mothers and Babies Research Centre Hunter Medical Research Institute New Lambton Heights New South Wales Australia; ^4^ Telethon Kids Institute University of Western Australia Perth Western Australia Australia

**Keywords:** ALSPAC, complex traits, grip strength, GWAS, neurodevelopment, Raine study

## Abstract

Grip strength (GS) is a proxy measure for muscular strength and a predictor for bone fracture risk among other diseases. Previous genome‐wide association studies (GWASs) have been conducted in large cohorts of adults focusing on scores collected for the dominant hand, therefore increasing the likelihood of confounding effects by environmental factors. Here, we perform the first GWAS meta‐analyses on maximal GS with the dominant (GSD) and non‐dominant (GSND) hand in two cohorts of children (ALSPAC, *N* = 5450; age range = 10.65–13.61; Raine Study, *N* = 1162, age range: 9.42–12.38 years). We identified a novel significant association for GSND (rs9546244, *LINC02465*, *p* = 3.43e−08*)* and replicated associations previously reported in adults including with a *HOXB3* gene marker that shows an expression quantitative trait locus (eQTL) effect. Despite a much smaller sample (~3%) compared with the UK Biobank we replicated correlation analyses previously reported in this much larger adult cohort, such as a negative correlation with coronary artery disease. Although the results from the polygenic risk score (PRS) analyses did not survive multiple testing correction, we observed nominally significant associations between GS and risk of overall fracture, as previously reported, as well ADHD which will require further investigations. Finally, we observed a higher SNP‐heritability (24%–41%) compared with previous studies (4%–24%) in adults. Overall, our results suggest that cohorts of children might be better suited for genetic studies of grip strength, possibly due to the shorter exposure to confounding environmental factors compared with adults.

## Graphical abstract

Manhattan plots for grip strength in the dominant (A) and non‐dominant (B) in a GWAS conducted in children. Top genes show association with both hands and replicate previous findings in large cohorts of adults.

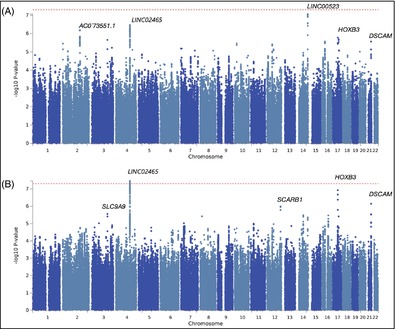

## 1 INTRODUCTION

Grip strength (GS) is an indicator of muscle fitness and has been shown to predict different health outcomes.^1^, ^2^, ^3^ In a longitudinal study on postmenopausal women (*N* = 2928) low GS—measured for the dominant hand—was associated with a slight increase of hip fractures (OR = 1.05, 95% CI: 1.01–1.09).^4^ Cheung et al.^5^ reported similar findings, where low GS scores (<1 SD from the mean) were associated with increased risk of major fractures in the Hong Kong population (OR = 1.24, 95% CI: 1.09–1.42; *N* = 4855). A longitudinal study on more than 1 million males found that GS was a reliable predictor of premature mortality, with stronger individuals presenting a lower risk for cardiovascular diseases, suicide and psychiatric disorders.^1^ Analysis in the UK Biobank found supportive evidence of a protective role of relative GS (calculated as the average mean of the right‐ and left‐hand measurements divided by body weight) for atrial fibrillation (OR = 0.75, 95% CI: 0.62–0.90) and coronary artery disease (CAD, OR = 0.69, 95% CI: 0.60–0.79).^6^ The authors also reported a strong negative genetic correlation between GS, depressive symptoms and attention deficit hyperactivity disorder (ADHD). A separate study in the UK Biobank showed that maximal grip strength is positively associated with cognitive measures of reasoning, prospective memory, number memory, visual memory and reaction time.^7^

In twin studies, GS heritability has been estimated in the range of 30%–65%.^8^, ^9^ To date, few studies have investigated the molecular genetics of GS. An initial genome‐wide association study (GWAS) conducted in two small Australian cohorts (*N*_total_ = 2629, age range 55–85) found no variants associated with maximal GS for the dominant hand.^10^ Analysis in the UK Biobank (*N* = 195,180, age range 40–69) identified 16 statistically significant associations and reported the SNP‐heritability (SNP‐h^2^) for maximal grip strength to be 24%.^11^ Statistically significant associations were located close to genes known to have a neuromuscular function (*ACTG1*) or implicated in rare monogenic syndromes characterised by progressive neurological/psychomotor impairment (*KANSL1* and *LRPPRC*). Associations were also detected in *HOXB3*, a highly conserved gene essential for morphogenesis and the establishment of the anterior–posterior axis.

A second GWAS in the UK Biobank (*N* = 223,315) on relative GS identified 64 associated markers.^6^ The lead variants were located close to genes involved in muscular development disease (myotonic dystrophy type 1, Pitt‐Hopkins syndrome, congenital heart defect and hand abnormalities, Char syndrome) and intellectual disability with a variant located in a long non‐coding RNA (*LINC01874*).

A recent meta‐analysis of categorical GWAS for GS (low vs. high) across 22 cohorts of elderly individuals (total *N* = 256,523 individuals aged 60 years and over, including 200,565 UK Biobank participants), identified 15 associated loci.^12^ Associated genes are involved in autoimmune disease (*HLA‐DQA1*) and osteoarthritis (*GDF5*, *ALDH1A2* and *SLC39A8*). Significant genetic correlations were found between low GS and osteoarthritis (*r*_g_ = 0.30), CAD (*r*_g_ = 0.19) and Alzheimer (*r*_g_ = 0.16). Exome sequencing analysis in the UK Biobank identified rare variants associated with GS in the *KDM5B*, *OBSCN*, *GIGYF1*, *TTN*, *RB1CC1* and *EIF3J* genes.^13^

Diet, sports activities and health conditions are expected to contribute to GS, potentially masking genetic effects. For example, analyses in younger participants, who accumulated less environmental exposures, might be more effective in identifying genes contributing to GS. Furthermore, we reasoned that the non‐dominant hand would be less influenced by training from everyday activities and therefore more likely to reveal genetic effects. Therefore, we conducted the analysis for grip strength testing both the dominant (GSD) and non‐dominant (GSND) hands. We report the first GWAS for two GS measures conducted in two cohorts of children (ALSPAC: *N* = 5450, mean age = 11.8 years; Raine Study: *N* = 1162, mean age = 10.6 years). Genetic correlation and polygenic risk score (PRS) analyses were performed for phenotypes, such as cardiovascular disorders, heel bone density and psychiatric disorders previously reported to be associated with GS in both behavioural and genetic levels.

## 2 METHODS

### 2.1 Cohorts

#### 2.1.1 ALSPAC cohort

Pregnant women resident in Avon, UK with expected dates of delivery between 1st April 1991 and 31st December 1992 were invited to take part in the Avon longitudinal study of parents and children (ALSPAC).^14^, ^15^ The initial number of pregnancies enrolled is 14,541. When the oldest children were approximately 7 years of age, an attempt was made to bolster the initial sample with eligible cases who had failed to join the study originally. The total sample size for analyses using any data collected after the age of seven is therefore 15,447 pregnancies. From age 7, all children were invited annually for assessments on a wide range of physical, behavioural and neuropsychological traits.

Grip strength was assessed at age 11 using the Jamar hand dynamometer, which measures isometric strength in kilogrammes. Before starting the test, the children were instructed on how to use the instrument and were given a practice trial. Children were encouraged to squeeze the dynamometer as long as possible. The test was repeated for each hand separately three times. The absolute highest score across all trials determined grip strength with the dominant hand (GSD). The maximal score for the other hand determined grip strength with the non‐dominant hand (GSND). For the current study, we removed individuals with missing phenotype or genotype data and outliers (outliers (*N* < 5) with very high grip strength scores of 65 and 42 kg), resulting in a total of 5450 participants entering the analyses (mean age = 11.8 years, SD = 0.24, *N*_males_ = 2674, *N*_Females_ = 2776; Table 1).

**TABLE 1:** Grip strength mean.

Sex	ALSPAC (SD) in kg			Raine Study (SD) in kg		
	*N*	GSD	GSND	*N*	GSD	GSND
Male	2674	20.8 (4.32)	18.7 (4.08)	609	17.0 (3.21)	15.17 (3.14)
Female	2776	19.6 (4.19)	17.6 (4.03)	553	15.44 (3.12)	13.59 (3.01)
Combined	5450	20.2 (4.29)	18.2 (4.09)	1162	16.26 (3.26)	14.42 (3.18)

#### 2.1.2 The Raine study

The Raine Study is a prospective pregnancy cohort of 2900 mothers recruited between 1989 and 1991 (https://rainestudy.org.au/).^16^ Recruitment took place at Western Australia's major perinatal centre, King Edward Memorial Hospital, and nearby private practices. Women who had sufficient English language skills, an expectation to deliver at King Edward Memorial Hospital, and an intention to reside in Western Australia to allow for future follow‐up of their child were eligible for the study.

The primary carers (Gen1) completed questionnaires regarding their respective study child, and the children (Gen2) had physical examinations at ages 1, 2, 3, 5, 8, 10, 14, 17, 18, 20 and 22.

Grip strength was assessed at Raine Study Gen2 10 follow‐up using the as part of the psychometric properties of the US developed McCarron Assessment of Neuromuscular Development (MAND).^17^ Grip strength was measured with a hand dynamometer, alternated twice on each hand. The skilled research assistant administering the assessment first demonstrated the task and instruction to ‘squeeze the handle as hard as you can’ was given prior to each attempt. Raw scores (measured in kilogrammes) were converted to scaled scores based on the participant's age. The absolute highest score across all trials determined grip strength with the dominant hand (GSD). The maximal score for the other hand determined grip strength with the non‐dominant hand (GSND). For the current study, we removed individuals with missing phenotype or genotype data, resulting in a total of 1162 participants entering the analyses (mean age = 10.59 years, SD = 0.18, *N*_males_ = 609, *N*_Females_ = 553; Table 1).

### 2.2 Genotyping, quality control and imputation

#### 2.2.1 ALSPAC cohort

Standard quality control (QC) and imputations were performed centrally by the ALSPAC team. Briefly, participants were genotyped using the Illumina HumanHap550 quad chip. The resulting raw genome‐wide data were subjected to standard QC methods. Individuals were excluded on the basis of gender mismatches; minimal or excessive heterozygosity and disproportionate levels of individual missingness (>3%). Population stratification was assessed by multidimensional scaling analysis and compared with HapMap II (release 22) European descent (CEU), Han Chinese, Japanese and Yoruba reference populations; all individuals whose genome did not cluster with the CEU reference were removed. SNPs with a minor allele frequency (MAF) of <1%, a call rate of <95% or evidence for violations of Hardy–Weinberg equilibrium (*p* < 5e−07) were removed. Cryptic relatedness was measured as the proportion of identity by descent (IBD > 0.1). Related subjects that passed all other quality control thresholds were retained during subsequent phasing and imputation. As a result, 9115 subjects and 500,527 SNPs passed these quality control filters and were imputed using Impute v3 and the HRC 1.1 reference panel (more information on the protocol followed by the ALSPAC team can be found here https://proposals.epi.bristol.ac.uk/alspac_omics_data_catalogue.html).

After imputation, SNPs with info score ≥0.4 and MAF ≥0.05 were retained for GWAS using BOLT‐LMM, leaving a total of 5,405,480 SNPs.

#### 2.2.2 The Raine study

The Raine Study Gen2 participants were genotyped on an Illumina 660 W Quad Array at the Centre for Applied Genomics, Toronto, Canada. QC of the GWAS genotyped data were performed as per standard protocol. In brief, a total of 1593 Raine Study Gen2 participants were genotyped on an Illumina 660 Quad Array, which included 657,366 genetic variants, consisting of ~560,000 single‐nucleotide‐polymorphisms (SNPs) and ~95,000 copy number variants (CNVs), at the Centre for Applied Genomics, Toronto, Canada. Plate controls and replicates with a higher proportion of missing data were excluded before individuals were assessed for low genotyping success (>3% missing), excessive heterozygosity, gender discrepancies between the core data and genotyped data, and cryptic relatedness (π > 0.1875, in between second‐ and third‐degree relatives—for example, between half‐siblings and cousins). At the SNP level, the SNP data were cleaned using plink^18^ following the Wellcome Trust Case–Control Consortium protocol.^19^ The exclusion criteria for SNPs included: Hardy–Weinberg‐Equilibrium *p* < 5.7 × 10^−7^; call‐rate <95%; MAF <1%; and SNPs of possible strand ambiguity (i.e., A/T and C/G SNPs). The cleaned GWAS data were imputed using MACH software^20^ across the 22‐autosomes and X‐chromosome against the 1000 Genome Project Phase I version 3.^21^ A total of 1494 individuals with 535,632 SNPs remained after genotype QC; imputation resulted in 30,061,896 and 1,264,4493 SNPs across the 22‐autosomes and X‐chromosome, respectively. Principal components (PCs) analysis was carried out, using SMARTPCA from v.3.0 of EIGENSOFT,^22^ on the cleaned genotyped data, where PCs were generated for purposes of adjusting for population stratification in all genetic analyses.

After imputation, SNPs with info score ≥0.4 and MAF ≥0.05 were retained for GWAS using ProbAbel, leaving a total of 5,982,053 SNPs across the 22 autosomes.

#### 2.2.3 Ethics approval

ALSPAC—Ethical approval for the present study was obtained from the ALSPAC Law and Ethics Committee and the Local Research Ethics Committees. Raine Study—Ethics approval for the original pregnancy cohort and subsequent follow‐ups were granted by the Human Research Ethics Committee of King Edward Memorial Hospital, Princess Margaret Hospital, the University of Western Australia and the Health Department of Western Australia.

#### 2.2.4 Consent to participate

ALSPAC—Informed consent for the use of data collected via questionnaires and clinics was obtained from participants following the recommendations of the ALSPAC Ethics and Law Committee at the time. Consent for biological samples has been collected in accordance with the Human Tissue Act (2004). Raine Study—Parents, guardians and young adult participants provided written informed consent either before enrolment or at data collection at each follow‐up. All research was performed in accordance with the approved guidelines.

#### 2.2.5 Heritability estimates and GWAS

The primary analysis was conducted in ALSPAC due to the sufficiently large sample size. We computed univariate SNP‐h^2^ with BOLT‐REML, which leverages raw data, implemented in BOLT‐LMM v2.3.4^23^, ^24^ using sex and age as covariates. Heritability in ALSPAC and the meta‐analysed GWAS results were also estimated with LDSC^25^ for direct comparisons with previous studies.

GWAS analysis was conducted in ALSPAC (*N* = 5450) with BOLT‐LMM v2.3.4^24^ under the standard infinitesimal linear mixed model (LMM) framework. As BOLT‐LMM implements linear mixed models, adjusting for PC was not needed.^26^ A recent study demonstrated that LMM that do not include PCs are a preferable option.^27^ Furthermore, the intercept scores from the LD‐score regression confirmed no evidence of population stratification (intercept_GSD_ = 1.01, intercept_GSND_ = 1.00).

As BOLT‐LMM requires a minimum sample of *N* ~ 5000, the GWAS analysis in the Raine Study (*N* = 1162) was conducted with Probabel v0.4.1^28^ under the standard linear model framework including the first two PCs as covariates. In both studies, sex and age were used as covariates on the basis of previously reported effects in behavioural analysis for grip strength conducted in the ALSPAC cohort.^29^ The genomic inflation factor (*λ*) was calculated with LDSC.

The results from the separate GWAS were meta‐analysed under inverse‐variance fixed‐analysis using METAL.^30^ Only markers available in both GWAS results were retained and were uploaded to FUMA to (i) identify markers previously reported in GWAS Catalogue, (ii) create regional plots for significant hits, (iii) run gene‐set enrichment analysis using MAGMA v1.6 and MsigDB v6.2, (iv) identify possible expression quantitative trait loci (eQTLs) for top markers (*p* < 1e−04) using GTEx v8 and (v) run gene‐based association using MAGMA v1.6.^31^ All results are reported in GRCh37 genome build.

#### 2.2.6 Replication analysis

The results from the meta‐analyses were used to test for replication of 93 SNPs associated with GS in the previous literature (*p* < 5e−08), which were selected as predefined lead SNPs in FUMA. The 93 lead SNPs were selected from ~800 SNPs that mapped at 89 risk loci (*r* ^2^ ≥ 0.6 to define independent variants, default setting). This resulted in an overall Bonferroni correction of 5.62e−04 (=0.05/89 risk loci).

#### 2.2.7 Genetic correlation analysis

LD score regression was applied to the summary statistics of the meta‐analyses to calculate the genetic correlation between GSD, GSND and traits previously reported to be associated to GS, namely schizophrenia (SCZ; Ref. [32]), bipolar disorder (BIP; Ref. [33]), overall fracture risk,^34^ ADHD,^35^ autism spectrum disorder (ASD; Ref. [36]), coronary artery disease (CAD; Ref. [37]), Alzheimer's disease (AD; Ref. [38]), heel bone mineral density (HBD, with right and left foot), and heart attack and grip strength with the right (grip.right.UKBB) and left (grip.left.UKBB) hand (http://www.nealelab.is/uk-biobank). We applied Bonferroni correction to account for multiple tests, resulting in a significance threshold of 0.05/(2 GS phenotypes × 12 associations tested) = 0.0021.

All analyses were restricted to the confidently imputed common SNPs from HapMap Phase III (1,025,494 SNPs), to avoid potential confounding effects arising from imputation quality.

#### 2.2.8 Gene‐based and gene‐set enrichment analysis

Gene‐based association analysis and Gene‐Set Enrichment Analysis (GSEA) were carried out in MAGMA (v.1.6) as implemented in FUMA (v1.3.5e). The MHC region was removed from the analysis. For the gene‐based analyses, SNPs were mapped against the 18,874 protein‐coding genes and associations were tested using multiple linear principal components regression for GSD and GSND. The Bonferroni‐corrected threshold used to determine statistical significance for gene‐based analysis was 0.05/18,874 = 2.65e−06. GSEA was conducted for all the pre‐defined gene set lists from Gene Ontology (GO).

#### 2.2.9 Polygenic risk score analysis

PRS analyses were performed with PRS‐CS^39^ for GSD and GSND in the ALSPAC cohort. Summary statistics were selected from GWAS that presented a SNP‐h^2^ >0.04, including SCZ, AD, BIP, overall fracture risk, ADHD, ASD, CAD and HBD right, HBD left. From the base GWAS datasets, SNPs with MAF >0.01 and INFO >0.9 were filtered and then clumped (LD *r* ^2^ ≥ 0.1 within a 250 kb window). Only overlapping SNPs between the base dataset, the data in ALSPAC and an external LD panel (1000 Genomes, Phase 3) were retained. Age, sex and 10 PCs were used as covariates in the analyses. The analysis was conducted with the default settings. A total of 9 base GWAS datasets entered the PRS analysis. We applied Bonferroni correction, resulting in a significance threshold of 0.05/(2 target phenotypes * 9 base GWAS) = 2.77e−03. Data preparation was performed with Python 3.9.

### 2.3 Data availability

The ALSPAC study website contains details of all the data that is available through a fully searchable data dictionary (http://www.bris.ac.uk/alspac/researchers/data-access/data-dictionary/). The data access policy is described here https://www.bristol.ac.uk/alspac/researchers/access/. The Raine Study website contains details of all the data that is available https://rainestudy.org.au/information-for-researchers/available-data/. The data access policy is described here https://rainestudy.org.au/information-for-researchers/project-application-process/.

All analysis code is available on Github (https://github.com/fabbondanza/grip_paper) and the full summary statistics are available at this link https://doi.org/10.17630/7588cd36-c612-4342-8e1b-3282707866a1.

Summary statistics were obtained from PGC summary statistics—https://www.med.unc.edu/pgc/results-and-downloads/; Neale's Lab UK Biobank summary statistics—http://www.nealelab.is/uk-biobank; Summary statistics for CAD—http://www.cardiogramplusc4d.org/data-downloads/.

## 3 RESULTS

### 3.1 The grip strength phenotypes

**FIGURE 1:**
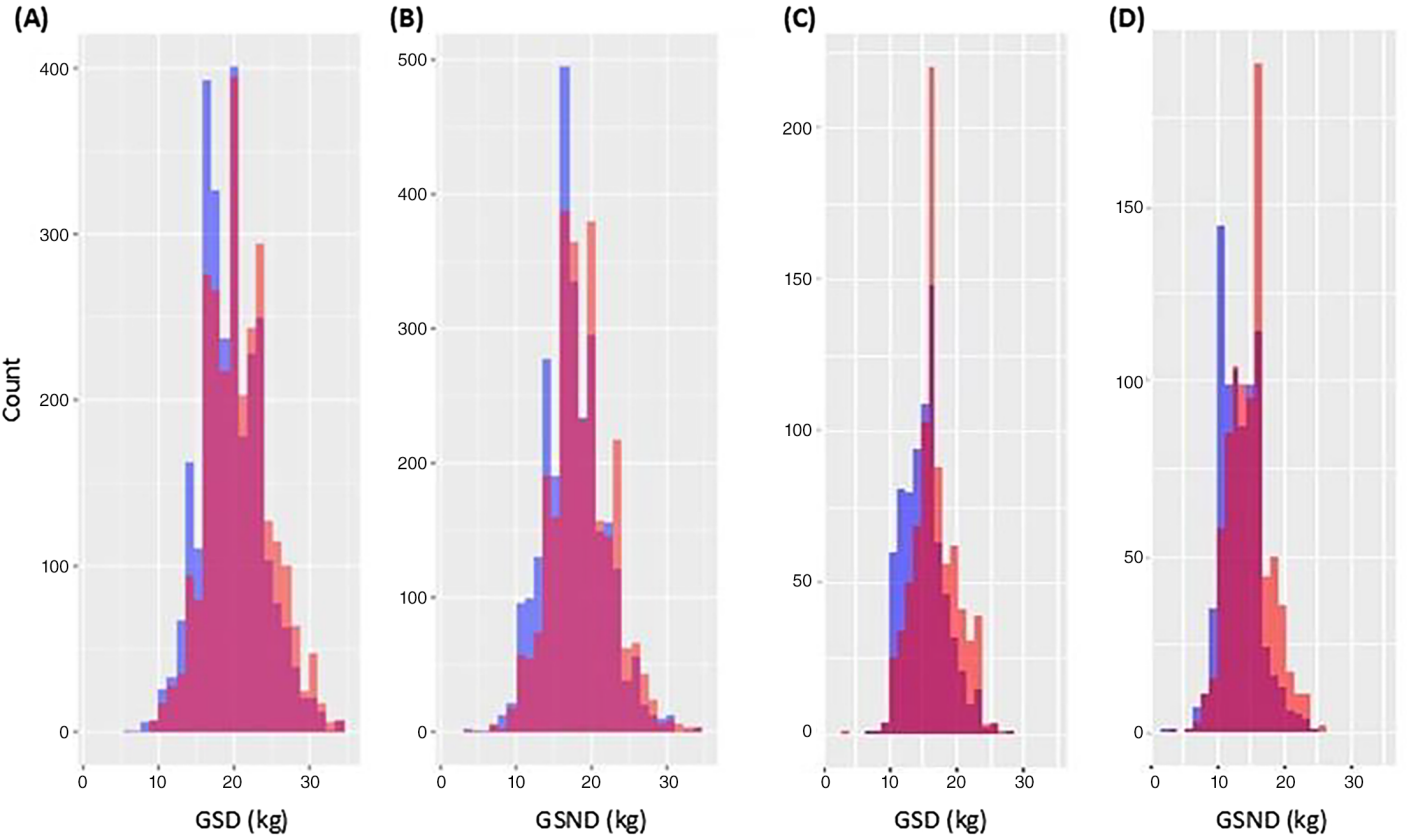
Distribution of grip strength with dominant (GSD) and grip strength with non‐dominant (GSND) scores stratified by sex in ALSPAC (A and B) and in the Raine Study (C and D). Females are represented in purple and males in orange.

GSD and GSND were normally distributed in both the ALSPAC and Raine Study cohorts (Figure 1). As expected from a previous study conducted in ALSPAC,^29^ males had higher scores than females (Table 1) and scores were positively correlated with age (ALSPAC: GSD *r*_(5454)_ = 0.14, *p* < 2e−16; GSND *r*_(5454)_ = 0.13, *p* < 2e−16; Raine Study: GSD *r*_(df=1160)_ = 0.097, *p* = 8.76E−04; GSND *r*_(df=1160)_ = 0.103, *p* = 4.41E−04; Figure S1). GSD and GSND were strongly correlated both in the ALSPAC (*r*_(5454)_ = 0.92, *p* < 2e−16) and in the Raine Study (*r*_(df=1160)_ = 0.87, *p* < 2.2e−16) cohorts.

### 3.2 Heritability estimates and genetic correlation

SNP‐*h* ^*2*^, computed with BOLT‐REML, was 0.393 (95% CI: 0.268–0.518) for GSD and 0.41 (95% CI: 0.288–0.532) for GSND in the ALSPAC sample. Estimates with LDSC, for a direct comparison with previous studies, were SNP‐*h* ^*2*^_GSD_ = 0.283 (95% CI: 0.095–0.471) and SNP‐*h* ^*2*^_GSND_ = 0.384 (95% CI: 0.184–0.584). Similar results were observed for LDSC estimates in the meta‐analysis of both cohorts: SNP‐*h* ^*2*^_GSD_ = 0.248 (95% CI: 0.097–0.399), SNP‐*h* ^*2*^_GSND_ = 0.299 (95% CI: 0.142–0.456). The SNP‐*h* ^*2*^ for the non‐dominant hand tended to be higher compared with the dominant with both methods, but confidence intervals overlapped.

GSD and GSND showed an almost perfect genetic correlation (*r*_g_ = 0.99, SE = 0.012) in ALSPAC using BOLT‐REML.

### 3.3 Genome‐wide association study

GWAS for GSD and GSND in the ALSPAC cohort (Tables S1 and S2; Figure S1) and the Raine Study (Figure S2 and Tables S3 and S4) analysed separately did not identified any statistically significant associations. No individual marker reached genome‐wide statistical significance.

Consistent with the higher SNP‐h^2^ observed for GSND compared with GSD with LDSC estimates, we observed that more than double the number of markers (434 v 197) showed *p* < 1e−04 for GSDN compared with GSD in the ALSPAC cohort.

**FIGURE 2:**
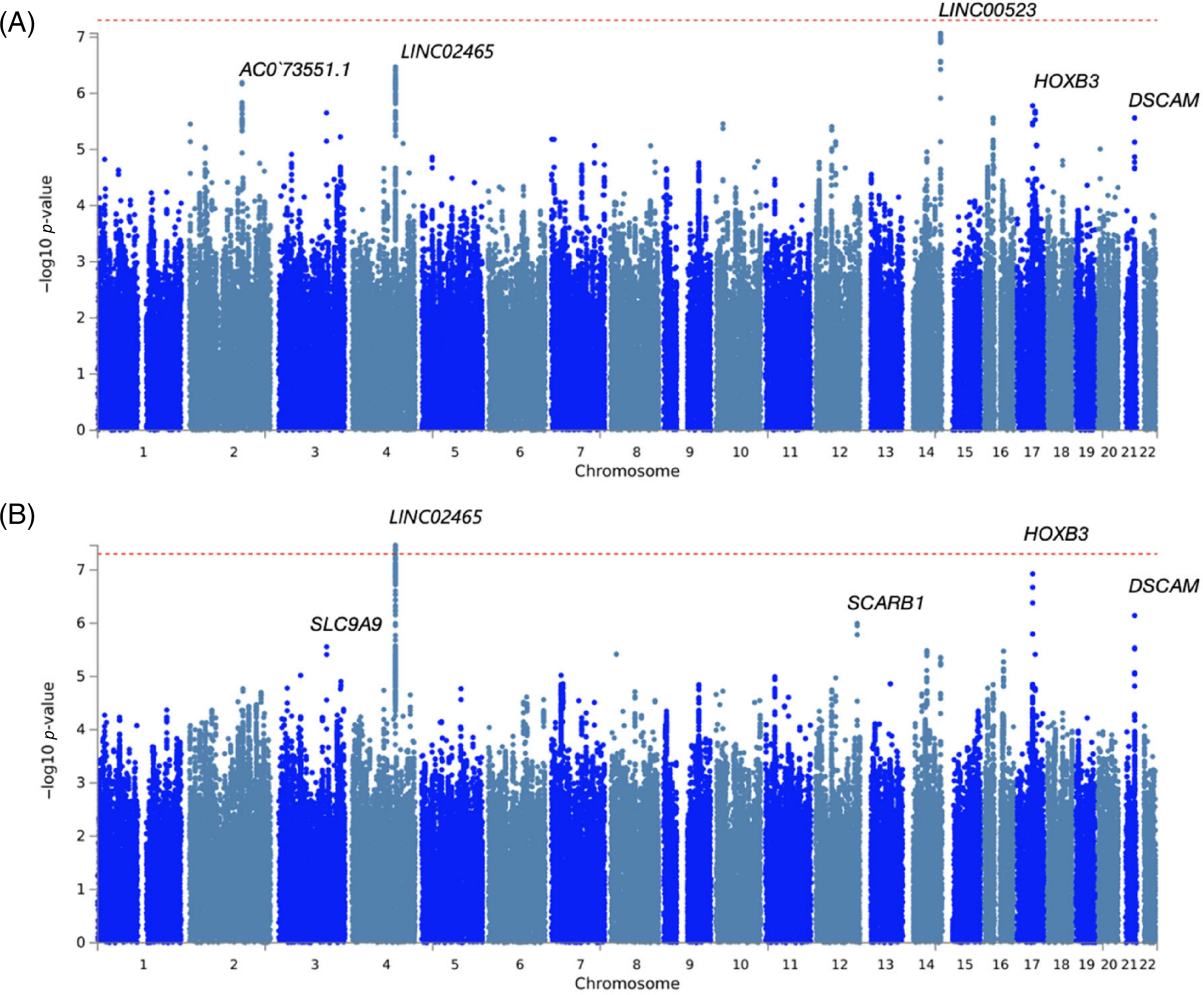
Manhattan plots for (A) grip strength with dominant and (B) grip strength with non‐dominant in the meta‐analysed sample. See Figure S3 for QQ plots and Figure S4 for the regional plot of the top associated locus at chromosome 4.

The ALSPAC summary statistics were meta‐analysed with the Raine Study summary statistics (Figure 2 and Table 2; Figures S4 and S5; Tables S5–S12). The genomic inflation factors in the meta‐analyses (*λ*_GSD_ = 1.040, *λ*_GSND_ = 1.044) suggested significant inflation of *p*‐values, but the intercept from LD‐score regression (intercept_GSD_ = 1.01, intercept_GSND_ = 1.00) suggested that this was due to polygenicity rather than population stratification (see QQ plot in Figure S3). The rs2968991 marker in *LINC02465* reached statistical significance for GSND and was among the top hits for GSD (Table 2; Figure S4). Markers among the strongest associations for both GSD and GSND included rs2555111 in *HOXB3*, rs13069868 in *SLC9A9* and rs66461687 in *DSCAM*.

**TABLE 2:** Top associated markers in the meta‐analyses.

Trait	Location	Marker	Closest Gene	MAF	Effect allele	Beta	*p*‐value	Allelic direction^a^
GSD	**14:101101813**	**rs78777886**	***LINC00523***	**0.06**	**T**	**−0.19**	**8.53e−08**	**−−**
GSD	**4:130878818**	**rs2968991**	***LINC02465***	**0.39**	**A**	**−0.08**	**3.38e−07**	**−−**
GSD^b^	2:156919650	rs111991299	*NBAS*	0.06	T	−0.17	6.43e−07	−**−**
GSD^b^	**17:46648899**	**rs2555111** ^c^	***HOXB3***	**0.47**	**T**	**0.08**	**1.66e−06**	**++**
GSD^b^	**17:54258240**	**rs6505044**	***ANKFN1***	**0.45**	**A**	**0.08**	**2.07e−06**	**++**
GSD^b^	**3:142972276**	**rs13069868**	***SLC9A9***	**0.26**	**T**	**−0.09**	**2.23e−06**	**−−**
GSD	**21:42084895**	**rs66461687**	***DSCAM***	**0.08**	**A**	**−0.15**	**2.74e−06**	**−−**
GSD^b^	16:27825858	rs1644605	*GSG1L*	0.40	T	0.08	2.75e−06	++
GSD^b^	10:21572854	rs971015	*LUZP4P1*	0.28	T	0.09	3.50e−06	++
GSD^b^	2:2905398	rs34838742	*AC019118.2*	0.19	T	0.10	3.53e−06	++
GSD^b^	12:49331240	rs11547436	*ARF3*	0.08	A	0.15	3.92e−06	++
GSND	**4:130878818**	**rs2968991**	***LINC02465***	**0.39**	**A**	**−0.09**	**3.43e−08**	**−−**
GSND^b^	**17:46648899**	**rs2555111** ^c^	***HOXB3***	**0.47**	**T**	**0.09**	**1.18e−07**	**++**
GSND	**21:42084895**	**rs66461687**	***DSCAM***	**0.08**	**A**	**−0.16**	**7.17e−07**	**−−**
GSND	12:125338529	rs10773112^c^	*SCARB1*	0.29	T	−0.09	1.01e−06	−**−**
GSND^b^	**3:142972276**	**rs13069868**	***SLC9A9***	**0.26**	**T**	**−0.09**	**2.78e−06**	**−−**
GSND	14:60530107	rs61994053^c^	*LRRC9*	0.13	T	0.11	3.27e−06	++
GSND^b^	16:58684945	rs151818^c^	*SLC38A7*	0.38	C	−0.08	3.36e−06	−**−**
GSND	8:18993252	rs112011071	*RP11‐1080G15.1*	0.06	A	0.17	3.79e−06	++
GSND^b^	**17:54259113**	**rs741128**	***ANKFN1***	**0.49**	**A**	**−0.08**	**3.85e−06**	**−−**
GSND	**14:101101462**	**rs79742354**	***LINC00523***	**0.06**	**A**	**−0.16**	**4.41e−06**	**−−**

*Note*: Loci reported in the top variants for both GSD and GSND are in bold.

^a^ Refers to the direction of the association for ALSPAC and Raine Study, respectively.

^b^ Refers to loci found to be associated with GS traits in previous literature (see Replication analysis and Table S14).

^c^ Refers to variants that showed eQTL effects in GTEx v8 (see Tables S7 and S11).

The rs2555111 variant in *HOXB3* showed a strong eQTL effect (lowest *p*‐value across multiple tissues: *p* = 1.40e−62), with each copy of the allele associated with higher grip strength (T allele), upregulating *HOXB‐AS1*, *HOXB2*, *HOXB3*, *HOXB6* and *HOXB7* across multiple tissues (for the full eQTL results, see Tables S7 and S11).

The top associated variants were annotated for previously reported GWAS associations, which included body mass index (*LINC02465*) and heel bone mineral density (*ANKFN1*) (Tables S8 and S12). We then conducted a replication analysis for 93 lead SNPs (Table S13) previously reported to be associated with grip strength. In total, 22 SNPs showed a statistically significant association corrected for multiple testing (*p* < 5.62e−04) (Table 2; Table S14). Two markers, rs2555111 in *HOXB3* and rs13069868 in *SLC9A9*, showed association with both GSD and GSND.

No significant results were found for gene‐based analyses and gene set enrichment analyses (GSEA) for GSD and GSND in the meta‐analysed data (Figure S5; Tables S6 and S10).

### 3.4 Genetic correlation and polygenic risk score analyses

**FIGURE 3:**
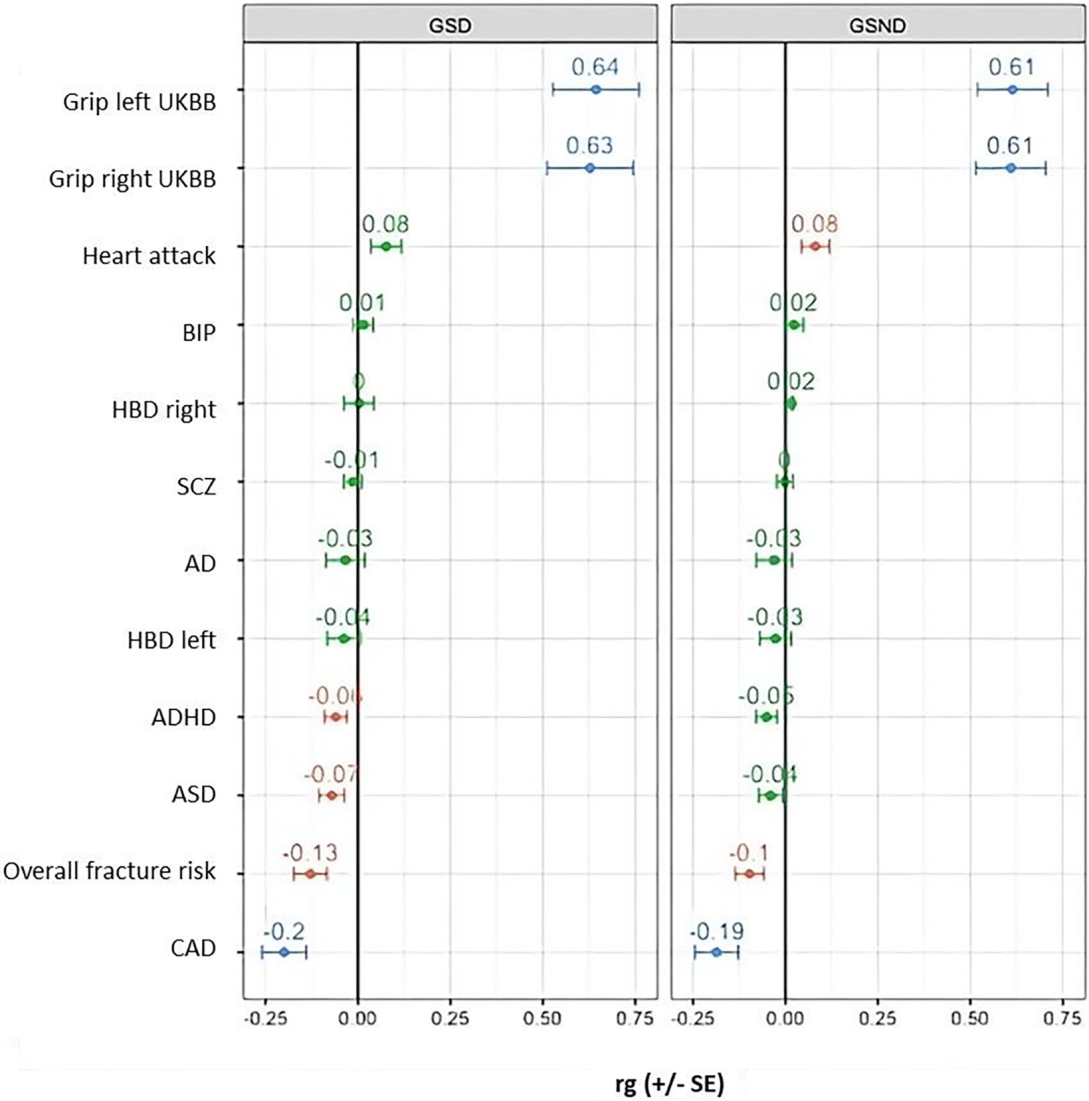
Genetic correlation (rg) analysis for grip strength with dominant (GSD) and grip strength with non‐dominant (GSND). The colour of the rg values refers to the *p*‐value with red indicating nominal significance (*p* < 0.05), green indicating a non‐significant result, and blue indicating statistical significance with *p* < 0.0021. AD, Alzheimer's disease; ASD, autism spectrum disorder; BIP, bipolar disorder; HBD.left, heel bone density with the left foot; Grip.left.UKBB, grip strength with the left hand from UK Biobank; Grip.right.UKBB, grip strength with the right hand from UK Biobank; HBD.right, heel bone density with the right foot; SCZ, schizophrenia.

Genetic correlations were tested for psychiatric (ADHD, BIP, ASD, SCZ, AD) and non‐psychiatric (heel bone density with the left [HBD left] and right foot [HBD right], CAD, heart attack, overall fracture risk, and the UK Biobank measures for grip strength with the right and with the left hand) traits (Figure 3; Table S15). We found a statistically significant positive correlation with the UK Biobank grip strength measures with both GSD and GSND (GSD—grip.left.UKBB: *r*_g_ = 0.64, *p* = 3.38e−08; GSD—grip.right.UKBB: *r*_g_ = 0.63, *p* = 6.72e−08, GSND—grip.left.UKBB: *r*_g_ = 0.61, *p* = 9.76e−11; GSND—grip.right.UKBB: *r*_g_ = 0.61, *p* = 9.51e−11). A negative correlation was found between high grip strength and CAD risk for both GSD (*r*_g_ = −0.2, *p* = 8e−04) and GSND (*r*_g_ = −0.19, *p* = 1e−04) that were consistent with previous reports for GS in an adult cohort (*r*_g_ = 0.19).^12^ Nominally significant correlations were observed for self‐reported history of heart attack (*r*_GSND_ = 0.081 [SE = 0.037], *p*_GSND_ = 0.03) and negative correlations with ADHD (*r*_GSD_ = −0.064 [SE = 0.030], *p*_GSD_ = 0.036), ASD (*r*_GSD_ *r* = −0.071 [SE = 0.033], *p*_GSD_ = 0.033) and overall fracture risk (*r*_GSD_ = −0.129 [SE = 0.045], *p*_GSD_ = 0.0037; *r*_GSND_ = −0.097 [SE = 0.039], *p*_GSND_ = 0.012).

We then conducted PRS analysis for the same set of traits after excluding heart attack due to the low SNP‐h^2^ (SNP‐h^2^ < 0.04) (Table S16). Although no association was statistically significant after correcting for multiple testing, we observed some nominally significant associations. Specifically, risk for ADHD was positively associated with grip strength (*p*_GSD_ = 0.017; *p*_GSND_ = 0.005) while risk for overall fracture risk showed a negative association (*p*_GSND_ = 0.017).

## 4 DISCUSSION

We report the first GWAS for grip strength measures in children. Previous genetic studies for GS were conducted predominantly in adult cohorts and focussed on measures of maximal strength, typically for the dominant hand. GS measures assessed in adults are likely to be confounded by environmental factors, linked to lifestyle, type of work or recreational activities with a stronger effect on the dominant hand due to more training.

Consistent with our reasoning, GSND showed a trend of higher SNP‐h^2^ compared with GSD when estimated with BOLT‐REML (0.39 v 0.41) and especially with LDSC (0.28 v 0.38; ALSPAC sample only). However, we note that the confidence intervals are overlapping and therefore it is not possible to reach firm conclusions. The difference in the latter case could have resulted from a lower number of SNPs for GSD compared with GSND passed the *p*‐value cut‐off to be included in the LDSC calculation. The SNP‐h^2^ observed in our study were higher compared with estimates observed in adults which ranged from 13%^6^ to 24%^11^ and were as low as 4% in older (>65 years old) individuals.^12^ Our results in combination with observations from the literature suggest that the heritability of grip strength decreases in older age due to environmental factors.^40^

Despite the smaller sample size of our study compared with analyses in adult cohorts, we both replicated previously reported loci and reported a novel statistically significant association, demonstrating the advantages of analysing cohorts of children. We anticipate that analyses in larger cohorts of children will lead to additional genetic associations. The novel association was detected for GSND at the *LINC02465* locus on chromosome 4. The *LINC02465* long non‐coding RNA has previously been found to be associated with body mass index and body size.^41^, ^42^, ^43^ The same locus ranked among the top associations also for GSD.

Different genes, including *LINC00523* (top association for GSD), *HOXB3*, *SLC9A9*, *ANKFN1* and *DSCAM* were among the top associations for both GSD and GSND. Given the strong correlation between GSD and GSND, such consistency it is not surprising, nevertheless it represents an internal control for our analyses. The reliability of our findings is further supported by the replication of 22 out of 93 associations previously reported in the literature, including for the *HOXB3*, *SLC9A9* and *ANKFN1* genes observed in top associations for both GSD and GSND.

The *HOXB3* association is of particular interest because it was underpinned by the same SNP (rs2555111), which also shows strong eQTL effects regulating multiple HOXB genes (e.g., *HOXB‐AS1*, *HOXB2*, *HOXB3*, *HOXB6* and *HOXB7*) across multiple tissues, including muscle‐skeletal tissue.

When testing for genetic correlations, risk for CAD showed a statistically significant signal consistent with what reported by Jones et al.^12^ in adults. Indeed, substantial statistically significant genetic correlation for GSD and GSND were observed with the grip strengths measures recorded for the adult participants of the UK Biobank.

Although not statistically significant, the PRS analysis showed a pattern of associations between risk for overall structures and low GS scores in line with associations observed at phenotypic level.^5^ The strongest association was observed between ADHD PRS and high GSND in opposite direction with what observed in the corresponding genetic correlation test. Further analysis in bigger samples is required before reaching any conclusions.

Indeed, a clear limitation of our study is its relatively small sample size. Analyses of grip strength measures in larger children's cohorts would have the statistical power to investigate the associations that we observed with neurodevelopmental traits like ADHD and the patterns that we reported for psychiatric traits including AD and BIP. In addition to a small sample size, another limitation is that our analysis was restricted to participants of European descendent. We hope that this work will be useful to inspire and design similar studies in more diverse populations.

In conclusion, by performing the first GWAS for grip strength in children, we both report novel findings as well as replicating associations previously reported in cohorts of adults but much larger in sample size. We hypothesise that analyses in children are less confounded and the availability of larger cohorts of children has the potential to advance our understanding of GS genetics which, in turn, might predict a number of health outcomes.

## CONFLICT OF INTEREST STATEMENT

The authors declare no conflict of interest.

## Supporting information


**Data S1.** Figures.

**Data S2.** Tables.

## Data Availability

The data that support the findings of this study are available from ALSPAC and the Raine study. Restrictions apply to the availability of these data, which were used under license for this study.
