# Supplementary material for: A GWAS for grip strength in cohorts of children—Advantages of analysing young participants for this trait
Source: Genes Brain Behav. 2024 Oct 8;23(5):e70003. doi: 10.1111/gbb.70003 (PMC11459231; doi:10.1111/gbb.70003)
Supplement: Supplementary file 1 — Data S1. Figures. [file GBB-23-e70003-s002.docx]

**A GWAS for grip strength in cohorts of children – advantages of analysing young participants for this trait.**

Filippo Abbondanza^1^, Carol A. Wang^2,3^, Judith Schmitz^1^, Krzysztof Marianski^1^, Craig E. Pennell^2,3^, Andrew J.O. Whitehouse^4^, Silvia Paracchini^1*^

*^1^ School of Medicine, University of St Andrews, St Andrews, Scotland*

*^2^ School of Medicine and Public Health, University of Newcastle, New South Wales, Australia*

*^3^ Hunter Medical Research Institute, New South Wales, Australia*

*^4^ Telethon Kids Institute, University of Western Australia, Western Australia, Australia*

*Correspondence to: sp58@st-andrews.ac.uk

**Supplementary Figures**


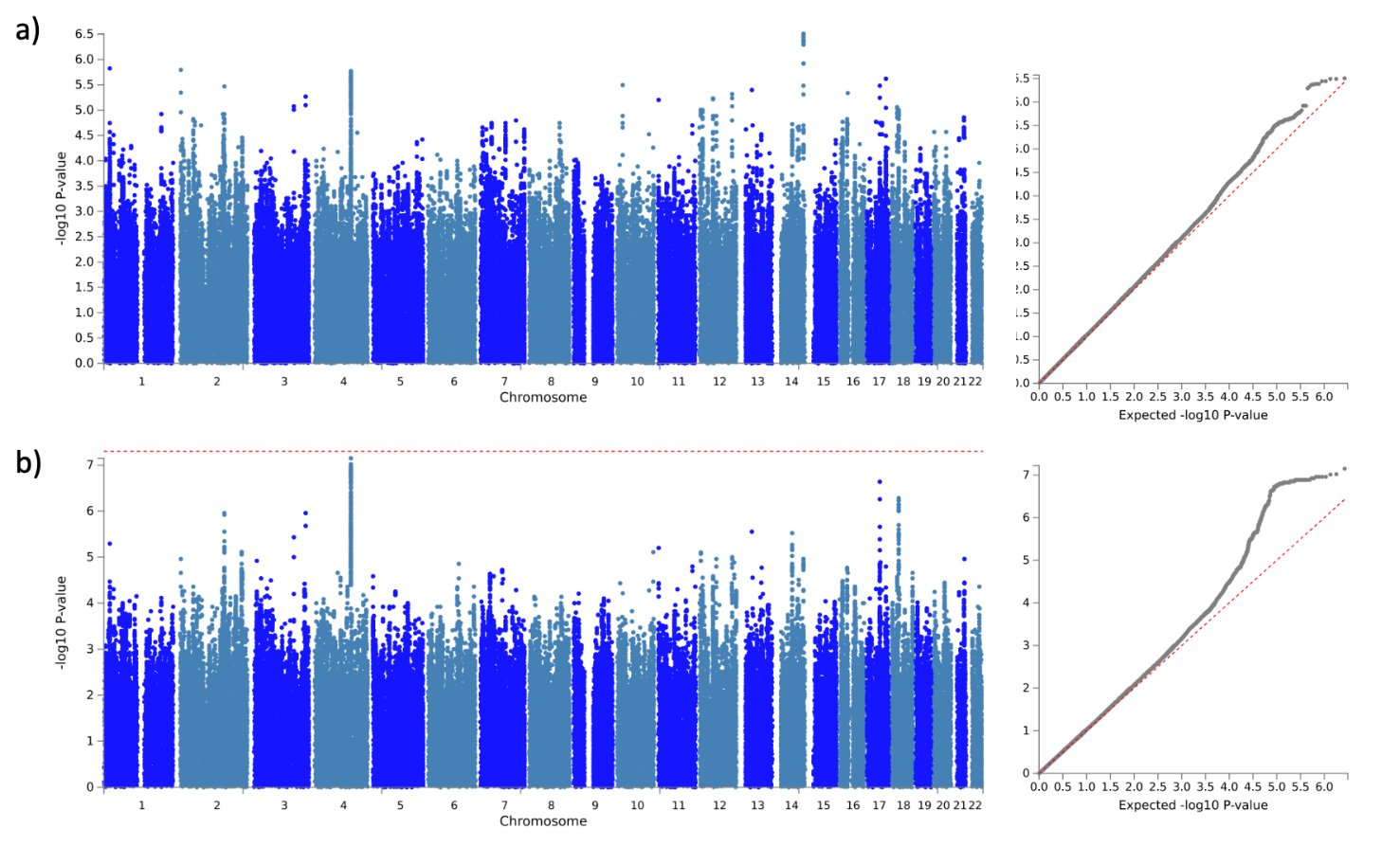
**Supplementary Figure S1. Manhattan and** QQ-plots for a) GSD and b) GSND in the ALSPAC cohort.


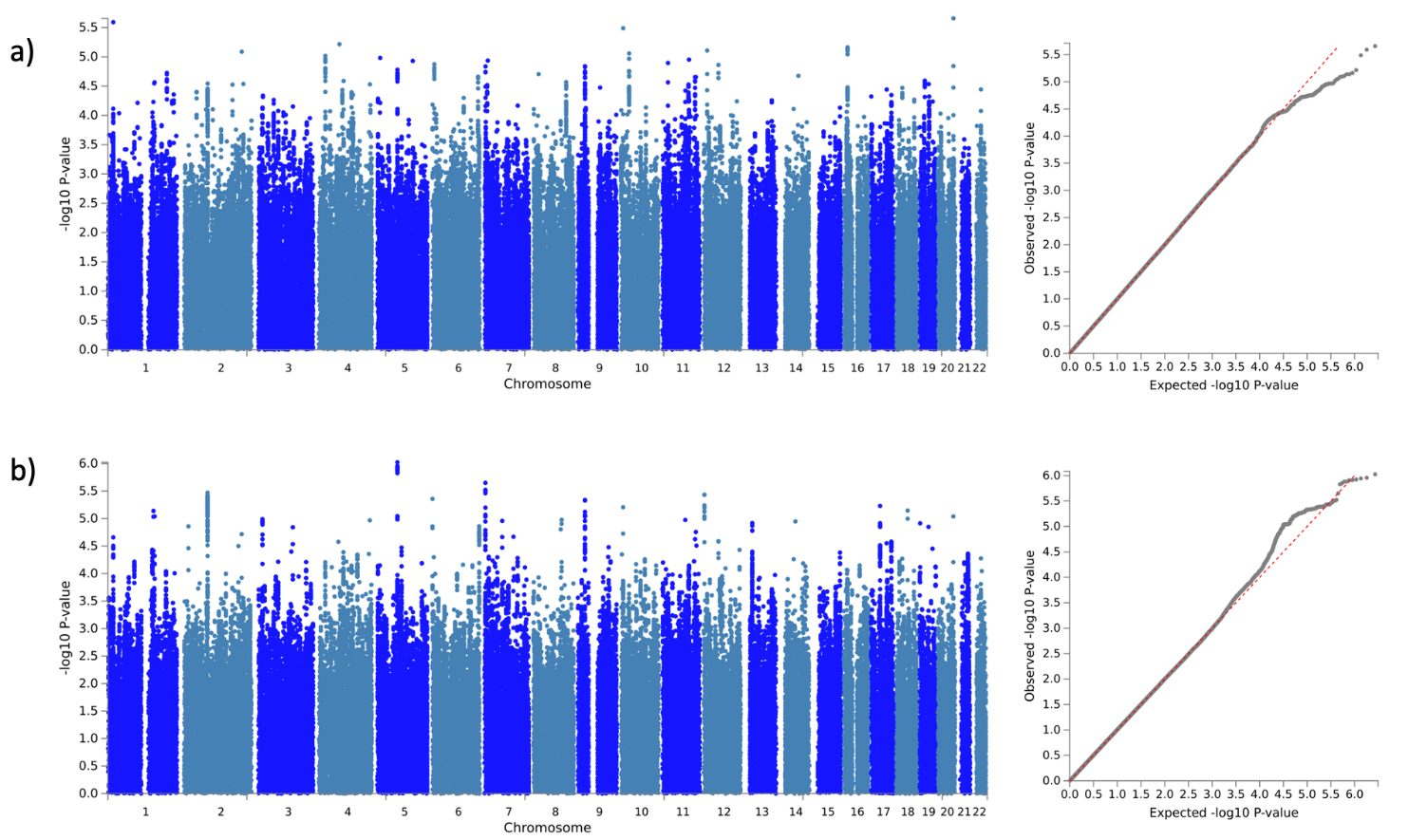


**Supplementary Figure S2:** GWAS and QQ-plot for a) GSD and b) GSND in the Raine Study


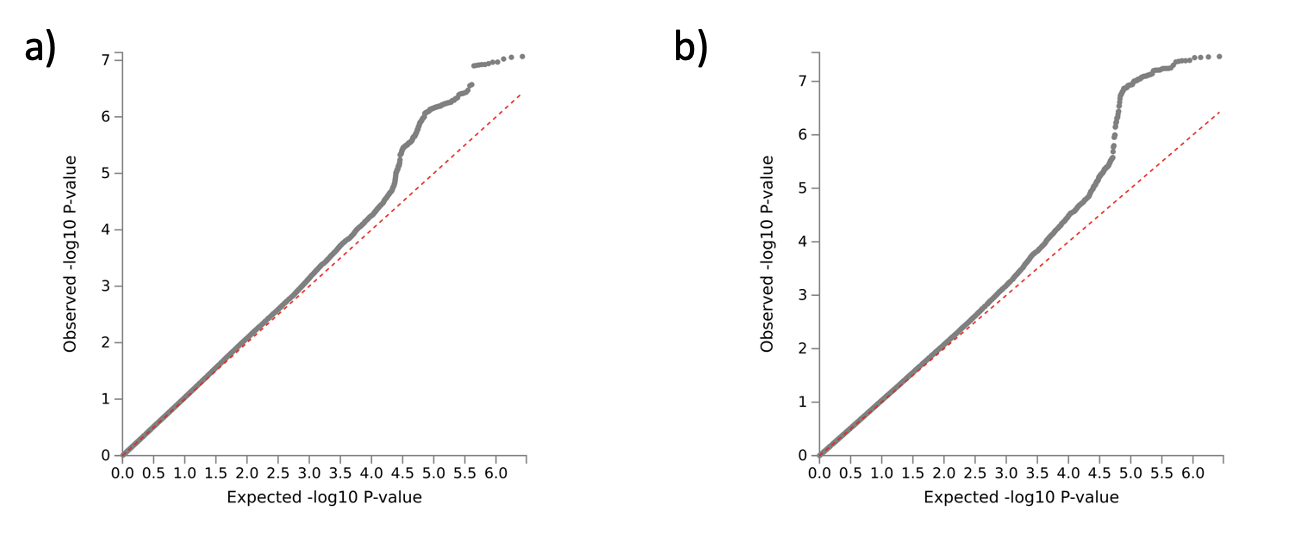


**Supplementary Figure S3:** QQ-plot for a) GSD and b) GSND in the meta-analysed sample.


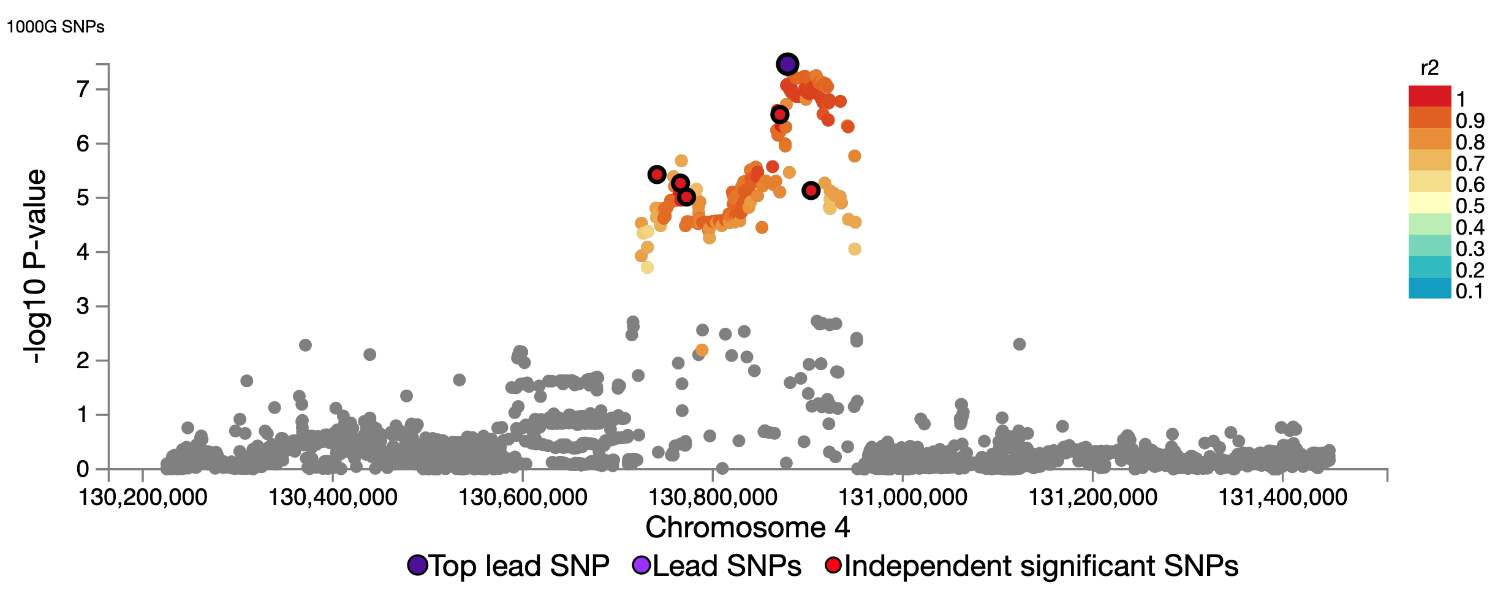


**Supplementary Figure S4:** Regional plot for rs2968991 in the GSND meta-analysis.


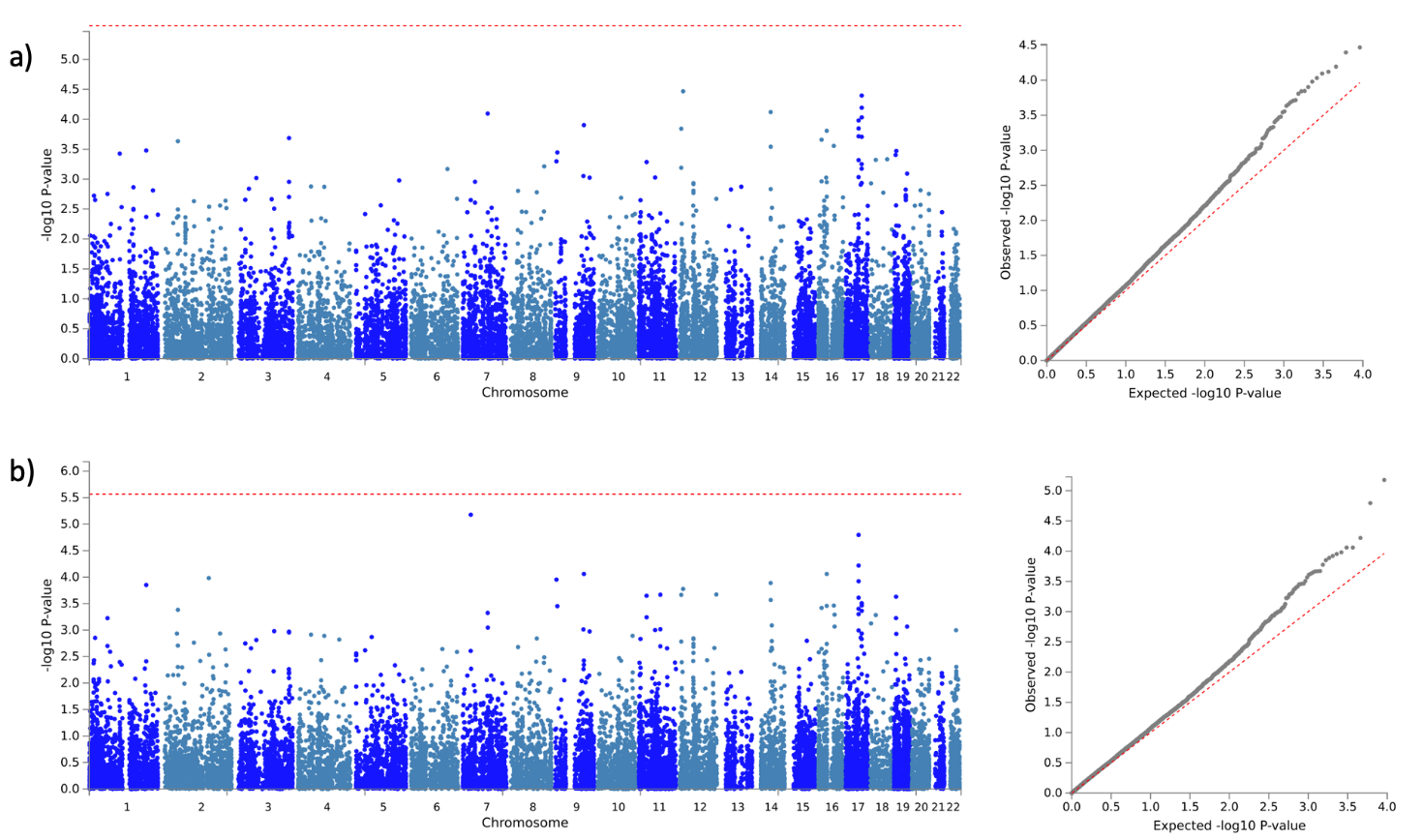
**Figure S5**: Gene-based meta-analysis Manhattan plots for a) GSD and b) GSND in the meta-analysed sample
